# Mesenchymal stem cells and their chondrogenic differentiated and dedifferentiated progeny express chemokine receptor CCR9 and chemotactically migrate toward CCL25 or serum

**DOI:** 10.1186/scrt310

**Published:** 2013-08-19

**Authors:** Mujib Ullah, Jan Eucker, Michael Sittinger, Jochen Ringe

**Affiliations:** 1 Tissue Engineering Laboratory & Berlin-Brandenburg Center for Regenerative Therapies, Department of Rheumatology and Clinical Immunology, Charité-Universitätsmedizin Berlin, Charitéplatz 1, 10117 Berlin, Germany; 2 Department of Hematology and Oncology, Charité-Universitätsmedizin Berlin, Charitéplatz 1, 10117 Berlin, Germany

**Keywords:** Chondrogenically differentiated cells, Stem cells, Cell migration, Chemotaxis, CCL25, CCR9

## Abstract

**Introduction:**

Guided migration of chondrogenically differentiated cells has not been well studied, even though it may be critical for growth, repair, and regenerative processes. The chemokine CCL25 is believed to play a critical role in the directional migration of leukocytes and stem cells. To investigate the motility effect of serum- or CCL25-mediated chemotaxis on chondrogenically differentiated cells, mesenchymal stem cells (MSCs) were induced to chondrogenic lineage cells.

**Methods:**

MSC-derived chondrogenically differentiated cells were characterized for morphology, histology, immunohistochemistry, quantitative polymerase chain reaction (qPCR), surface profile, and serum- or CCL25-mediated cell migration. Additionally, the chemokine receptor, CCR9, was examined in different states of MSCs.

**Results:**

The chondrogenic differentiated state of MSCs was positive for collagen type II and Alcian blue staining, and showed significantly upregulated expression of *COL2A1*and *SOX9*, and downregulated expression of CD44, CD73, CD90, CD105 and CD166, in contrast to the undifferentiated and dedifferentiated states of MSCs. For the chondrogenic differentiated, undifferentiated, and dedifferentiated states of MSCs, the serum-mediated chemotaxis was in a percentage ratio of 33%:84%:85%, and CCL25-mediated chemotaxis was in percentage ratio of 12%:14%:13%, respectively. On the protein level, CCR9, receptor of CCL25, was expressed in the form of extracellular and intracellular domains. On the gene level, qPCR confirmed the expression of *CCR9* in different states of MSCs.

**Conclusions:**

CCL25 is an effective cue to guide migration in a directional way. In CCL25-mediated chemotaxis, the cell-migration rate was almost the same for different states of MSCs. In serum-mediated chemotaxis, the cell-migration rate of chondrogenically differentiated cells was significantly lower than that in undifferentiated or dedifferentiated cells. Current knowledge of the surface CD profile and cell migration could be beneficial for regenerative cellular therapies.

## Introduction

Human mesenchymal stem cells (MSCs) hold great promise for tissue regeneration. During tissue repair, MSCs migrate to the sites of injury and participate in the repair process [1,2]. Stem cell migration not only plays a potential role in cell colonization inside biomaterial scaffolding [3], but also takes part in the reorganization of matrix [4]. Moreover, the guided migration of MSCs creates a therapeutic environment for bone regeneration [5]. These features emphasize the importance of targeted stem cell migration in tissue-engineering approaches.

Stem cell migration improves the curative ability of diseased tissues via appropriate homing inside injured sites [1,2,6]. Previously, it was reported that cell migration and subsequent suitable colonization of progenitor stem cells within injured sites accelerate myocardial regeneration [7,8], reduce heart damage [9,10], aid in recovery from spinal-cord injuries [11], cure nerve damage [12], and repair cartilage [13,14]. The MSCs have the potential to migrate through bone marrow endothelium, by using the regulatory mediators of matrix metalloproteinase-2 and tissue-inhibitor metalloproteinase-3 [15]. The administration of allogenic MSCs, whether derived from bone marrow or from adipose tissue, was reported for cellular proliferation, neurogenesis, and takes part in the functional recovery of brain after ischemic stroke [16]. Moreover, clinical trials of using human MSCs for bone fractures, bone defects, and cartilage disorders have been performed [17-19]. The investigation of targeted stem cell migration could be beneficial for tissue regeneration, especially for cartilage restoration. Chondrocytes in the articular cartilage lack innervations and vascularization and have low mitotic potential. Moreover, the chondrocytes have no physical contact to each other and entrapped into extracellular matrix [20-22]. These features make cartilage restoration is a hot issue in case of regeneration.

Autologous chondrocyte transplantation is an established technique for cartilage repair [23-25], which consists of chondrocyte isolation, *in vitro* dedifferentiation, and transplantation [25-27]. It is established that dedifferentiation is necessary to achieve a high cell number, and it is considered a curative step in such technologies [28-30]. However, massive dedifferentiation of chondrocytes results in loss of the chondrogenic phenotype and formation of primitive multipotent cell types [28-31]. To overcome such shortcomings, chondrogenic maintenance cues such as cytokines, chemokines, and growth factors are required to regulate and control the process of chondrocyte transplantation. The theoretic assumption is that this would increase remedial time and therapeutic cost because of *in vivo* posttransplantational procedures for chondrogenic differentiation and maintenance. It necessitates the use of such culture techniques and cell types, which not only maintain a chondrogenic-specific phenotype, from the beginning of transplantation, but also proliferate to increase the number of cells.

Therefore, the direct mobilization of endogenous cells and subsequent migration to the point of injury could be a promising approach for cartilage regeneration. In this context, the motility and migratory features of chondrocytes have been characterized [32]. To investigate the migratory effect of serum- or CCL25-mediated chemotaxis on chondrogenic cells, we isolated differentiated cells from compact pellets, after 28 days of chondrogenic differentiation. They maintained the chondrogenic nature for about 14 days in the culture and were able to proliferate. After chondrogenic confirmation, their surface profile and cell-migration ability were examined for serum- or CCL25-mediated chemotaxis.

Present strategies of stem cells transplantation advocate the use of MSCs [23,33-35], for diverse regenerative application, including cartilage repair [23,26]. In some cases, the clinical use of MSCs is considered more valuable than autologous chondrocytes transplantation [36,37], as it requires one less knee surgery, is easy to isolate, has a high proliferative rate, reduces cost, and provides better regenerative efficiency [28,35,36]. For instance, the use of magnetized MSCs is the best choice for articular cartilage repair [38]. In such cases, one controversial and basic question needs an answer: which cell type would be more suitable for cartilage regeneration, undifferentiated MSCs or their chondrogenic differentiated progeny? Therefore, we investigated the cell-migration profile of chondrogenically differentiated cells compared with the undifferentiated and dedifferentiated states of MSCs, according to already described formulation and concentration of allogenic serum [39].

However, allogenic serum has a complex composition [40-42], which is unknown and undefined for some molecular functions. It emphasizes the need for a defined and targeted chemokine, to make the present regenerative strategies more valuable and beneficial for appropriate cell homing. Moreover, chemokines are recognized as an essential factors for diverse cellular process including activation of the central hub of cellular migration via direct or indirect mechanisms and signaling events [39,43-45], and stimulation of the therapeutic efficiency of regeneration.

Chemotaxis is defined as directional movement of cells toward concentration gradients or chemoattractants, whereas chemokinesis is random cell movement without any chemoattractants [46]. Directional migration of MSCs to the site of injury is controlled by several factors, such as hypoxia and the Rho family of GTPases [47,48]. Generally, tissue regeneration requires a coordinating and well-regulating cell migration for its restoration in response to different cues like cytokines and growth factors [43,49]. Apart from this, chemokines play a vital role in a biologic plethora of migration and are considered guided cues for directional and targeted stem cell trafficking [39,43,49]. Chemokines enable the activity of migratory processes in hematopoietic and nonhematopoietic cells [50], navigate the cellular trafficking between tissue compartments, and play a potential role in cell activation, differentiation, survival, and recruitment of leukocytes [51]. In addition, they play a decisive role in mobilization of T lymphocytes during allergenic reactions [52] and contribute to the complex pathophysiology of asthma by using the coordinating network of cellular activation and signaling web [53].

Chemokine-based recruitment of MSCs to the point of injury is a promising approach, whereas chemokine (C-C motif) ligand 25 (CCL25) could play a vital role in cell migration [44,54]. After nerve damage or myocardial infarction, the mutual interactions of chemokines and their receptors mediate the migration of MSCs to injured sites [55]. Obviously, to understand the underlying mechanism would be of interest. In this context, CCL25 has been suggested as a potential chemoattractant for the directional movement of MSCs [56], and C-C chemokine receptor type 9 (CCR9) is known as a cognate receptor of CCL25 [57,58]. To check whether the chondrogenic differentiated state of MSCs affects the cell-migration rate, we performed the chemotaxis assay for undifferentiated, chondrogenic differentiated and dedifferentiated cells, by using the chemokine CCL25 [54]. Furthermore, the receptor CCR9 was examined in different states of MSCs, as CCR9 is an established known receptor of CCL25 and plays a decisive role in the targeted migration of stem cells [43,44,54].

To cope with the challenges of the growing tissue-engineering industry, we need an appropriate cell source and suitable cell types, which are able not only to migrate to the site of injuries or damage in a well-guided way, but also to facilitate quick regeneration. Our introduced cell types could be valuable and beneficial in this regard.

## Materials and methods

### Ethics statement and MSC isolation

The study was approved by the institutional ethical committee of the Charité-University Medicine Berlin. Written informed consent was obtained from all participants, as a requirement of the ethical review board. The human MSCs were isolated from iliac crest bone marrow aspirates (*n* = 3; two men, one women; average age, 52.3 ± 1.5 years) of the healthy donors, who were examined to exclude hematopoietic neoplasms and were histologically diagnosed as normal. The 1-ml aspirate was seeded per T175 cm^2^ of culture flasks (Becton Dickinson, Heidelberg, Germany). After 72 hours, nonadherent cells and cellular debris were washed out by media exchange, and cultures were further expanded in Dulbecco Modified Eagle Medium (DMEM; Biochrom, Berlin, Germany), supplemented with 10% fetal bovine serum (FBS; Hyclone, Cramlington, UK), 20 m*M* Hepes buffer (Biochrom), 2 m*M* L-glutamine (Biochrom), 2 ng/ml human basic-fibroblast growth factor (bFGF; Pepro Tech, London, UK) 100 units/ml penicillin and 100 μg/ml streptomycin (Biochrom), under established conditions. After expansion and subsequent confluences, the cells were detached with trypsin (0.05% 1 m*M* EDTA), and replated until passage 3.

### Chondrogenic differentiation

For chondrogenic differentiation, 2.5 × 10^5^ MSCs were centrifuged (150 *g*, 5 minutes) to form high-density micromass culture pellets. The chondrogenic differentiation of these pellets was achieved for 28 days with DMEM (4.5 g/L glucose; Biochrom), ITS supplements, 100 n*M* dexamethasone, 0.17 m*M* ascorbic acid-2-phosphate, 1 m*M* sodium pyruvate, 0.35 m*M* L-proline (all Sigma-Aldrich) and 10 ng/ml transforming growth factor-β3 (TGF-β3; PeproTeck, Hamburg, Germany). The control pellets were cultured in the same medium in the absence of TGF-β3. The medium (500 μl) was changed 3 times per week.

### Cell isolation from chondrogenic pellets and dedifferentiation

After chondrogenic differentiation of the pellets for 28 days, cells were isolated with 300 U of collagenase II, 20 U of collagenase P, and 2 m*M* CaCl_2_ for 90 minutes at 37°C [59]. Subsequently, some cells were cultured in a monolayer for 14 days in the presence of the chondrogenic differentiation-specific stimulus of TGF-β3, to maintain their chondrogenic nature. Conversely, the chondrogenically differentiated cells were cultured for five passages in the normal MSC expansion medium to accelerate proliferation and to generate dedifferentiated progenitor cells.

### Flow-cytometric analysis for cell-surface screening

Fluorescence-activated cell sorting (FACS) analysis was performed not only to characterize the MSCs for their typical specific surface antigens, but also to determine the expression of these antigens in chondrogenic differentiated and dedifferentiated cells. For all experimental cell types, the cells (*n* = 3) were prepared in the form of a single-cell suspension, then washed with PBS/0.5% bovine serum albumin (BSA; both Biochrom), and centrifuged for 5 minutes at 250 *g*. The resuspended cells in the cold PBS/0.5% BSA were incubated for 15 minutes on ice with R-phycoerythrin-labeled mouse anti-human CD14, CD34, CD73, CD166, and fluorescein isothiocyanate (FITC)-labeled mouse anti-human CD44, CD45, CD90, and CD105 antibodies. All antibodies were purchased from BD-Pharmingen (Heidelberg, Germany) except CD105, which was purchased from Acris Antibodies (Hiddenhausen, Germany).

After incubation, the cells were centrifuged (250 *g*, 5 minutes), washed with cold PBS/0.5% BSA, and resuspended in the same buffer before cytometric analysis. To examine the extracellular domain of CCR9 receptor, staining was performed as described earlier but with PE-labeled mouse anti-human CCR9 (R&D Systems, Wiesbaden, Germany). For measurement of the intracellular domain of the CCR9 receptor, one additional step of permeabilization was added. After fixation with 4% paraformaldehyde (Sigma, Germany) for 15 minutes, cells were permeabilized for 10 minutes with FACS permeabilizing solution-2 (Becton Dickinson, Germany) and then processed as described earlier. The propidium iodide (100 μg/ml; Sigma-Aldrich) staining was applied for the exclusion of dead cells and cellular debris, whereas unstained cells were used as a negative control. The single-cell suspension was analyzed with flow cytometry, and CellQuest software (Becton Dickinson) was used for the interpretation and analysis of results.

### Migration potential of cells

Migration potential of MSCs, chondrogenic differentiated cells, and dedifferentiated cells were assessed in response to 10% human allogenic serum or CCL25 chemokine (PeproTech, Germany). An already established chemotaxis assay by our group [33,54,60], was performed for all cell types with 8-μm pore size polycarbonate membranes in 96-multiwell format ChemoTx plates (Neuroprobe, Gaithersburg, USA). For migration of cells, either 10% allogenic serum or a selected concentration of CCL25 (500 nm, 750 nm, and 1,000 nm) [54] was applied in triplicate to the lower wells. The 4 × 10^4^ cells for serum and 3 × 10^4^ cells for CCL25 were seeded in the upper wells and incubated for 20 hours at 37°C. Negative controls were performed without chemokine or serum. Migrated cells were fixed in methanol/acetone, stained with hemacolor (Merck, Germany), and counted microscopically with Image J software.

### Histology and immunohistochemistry

To examine chondrogenesis, the high-density micromass pellets were embedded in Tissue-Tek with O.C.T compound (Sakura Finetek, Torrance, USA), and then were frozen in liquid nitrogen and cryosectioned (6-μm thickness). For the cartilage-specific proteoglycan examination, these sections were stained with Alcian blue 8GX (Roth, Karlsruhe, Germany) and counterstained with nuclear fast red (Sigma Aldrich). For the deposition and accumulation of collagen type II in the ECM, cryosections (6 μm) were incubated for 1 hour with primary rabbit anti-human type II collagen antibodies (Acris Antibodies). Subsequently, the sections were processed according to the manufacturer’s recommendation with the Envision system peroxidase kit (DAKO, Hamburg, Germany), followed by hematoxylin counterstaining (Merck, Darmstadt, Germany). The stained sections in the control samples were prepared from the chondrogenic control pellets.

To stain the chondrogenic differentiated and dedifferentiated cells, 2 × 10^5^ cells were transferred to each well of the four-well chamber slides (Thermo-scientific, Germany). The cells inside chamber slides were cultured for 3 days under standard conditions to ensure the proper attachment of the cells to the slide surface. For direct staining, the cells were fixed for 5 minutes in already cooled 3.7% formaldehyde in PBS. Subsequently, cells were stained according to the standard procedure, as described for cryosections of the chondrogenic pellets.

### RNA isolation and qPCR

For RNA isolation, the MSCs, chondrogenic differentiated, and dedifferentiated cells were mixed with TriReagent (Sigma-Aldrich). While the differentiated chondrogenic pellets [11-17], were first pooled for each individual donor (n = 3) in the 2-ml Eppendorf tube, then the TriReagent was mixed with these pellets and mechanically homogenized with an Ultra-Turrax (IKA, Staufen, Germany). Then 1-bromo-3-chloro-propane (Sigma-Aldrich) was added to all samples, followed by centrifugation (45 minutes, 13,000 *g*), and the upper phase, being free of proteins, was collected and mixed with an equal amount of ethanol. Subsequently, samples were processed with the RNeasy Mini Kit (Qiagen, Hilden, Germany), according to manufacturer recommendation. The quantity and quality of eluted RNA was ensured with NanoDrop measurements (NanoDrop Products, Wilmington, USA).

For qPCR, cDNA was synthesized from 2.5 μg total RNA by using the iScript cDNA synthesis kit (BioRad, Munich, Germany). The TaqMan qPCR was executed in triplicates in 96-well optical plates on a Mastercycler ep Realplex2 S system (Eppendorf, Hamburg, Germany). The gene-expression assays for typical chondrogenic-specific genes was performed with TaqMan probes and primer sets (Applied Biosystems, Darmstadt, Germany). Quantitative gene expression was analyzed for collagen type 2 A1 (*COL2A1*; Hs 00264051_m1), SRY (sex*-*determining region Y)-box-9 (*SOX9*; Hs 00165814_m1), C-C chemokine receptor type 9 (*CCR9*; Hs 01890924_s1), and glyceraldehyde-3-phosphate dehydrogenase (*GAPDH;* Hs99999905_m1). The expression of *COL2A1* and *SOX9* genes was normalized to the endogenous *GAPDH* expression level and calculated with the 2-ΔΔCt formula in percentage of *GAPDH* expression [61].

### Statistical analysis

The statistical analysis was performed by using SigmaStat 3.5 software (Systat Software, USA), whereas GraphPad Prism4 (GraphPad Software) was used for drawing graphs. Simple Student *t* test was used for statistical assessment, and asterisks were assigned in the order P* < 0.05, P** < 0.01, and P*** < 0.001 for statistically significant values, whereas exact *P* values were mentioned for statistically nonsignificant data sets. Error bars in all figures represent standard error of the mean.

## Results

### MSC isolation, authentication, and chondrogenic differentiation

**Figure 1:**
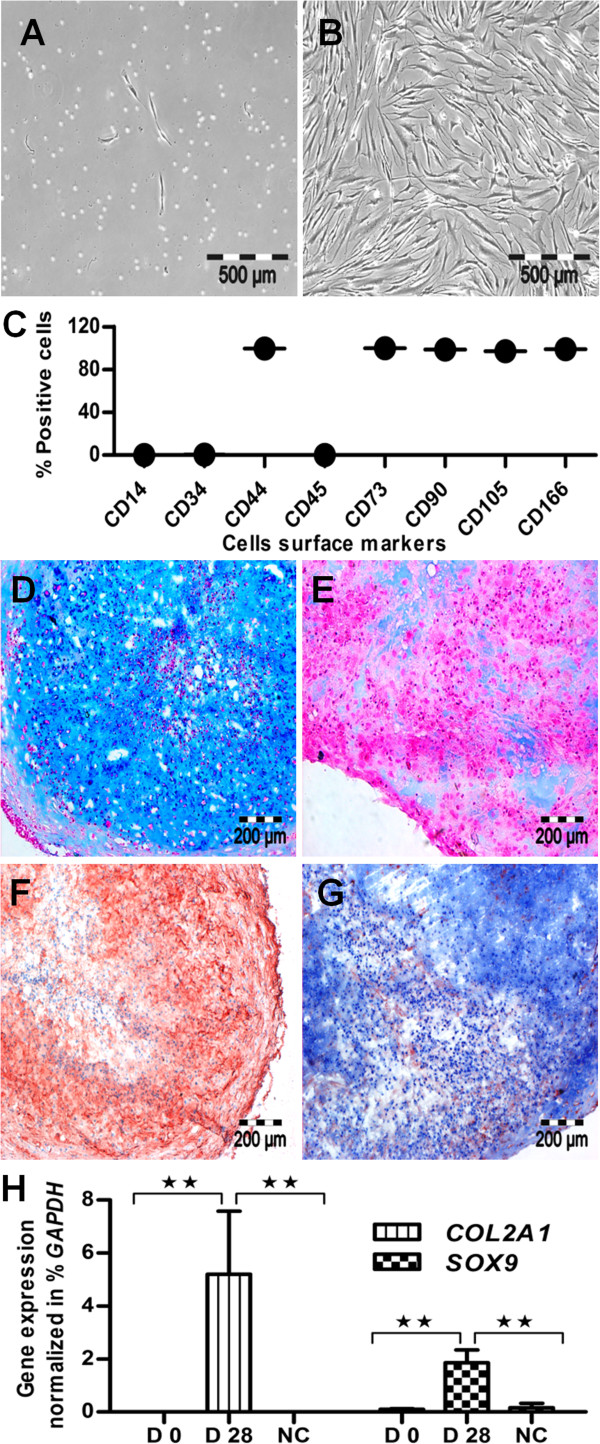
**MSC isolation, confirmation, and chondrogenic differentiation. (A)** MSCs appeared as single cells in P0, and **(B)** showed uniform growth and fibroblast-like morphology in P3. **(C)** Flow-cytometric analysis revealed positive expression for typical MSC antigens like CD166, CD105, CD90, CD73, and CD44, but negative expression for hematopoietic lineage-specific antigens like CD45, CD34, and CD14 (three biologic replicates; *n* = 3), mean ± SEM. **(D)** On chondrogenic differentiation, they showed positive expression for Alcian blue staining compared with **(E)** control. **(F)** Similarly, they were positive for collagen type II expression compared with **(G)** unstimulated samples. **(H)** On the gene level, the chondrogenic differentiation was confirmed by significantly upregulated expression of *COL2A1* and *SOX9* genes compared with negative controls and undifferentiated MSCs, day 0 (*n* = 3). Student *t* test was performed for statistical analysis, and asterisks were assigned in the order P* < 0.05, P** < 0.01, and P*** < 0.001; mean ± SEM. Bar A, B, 500 *μ*m; D through G, 200 *μ*m.

Human MSCs were isolated from bone marrow aspirates, as shown in the form of individual longitudinal cells in P0 (Figure 1A), which became homogeneous with subsequent growth and revealed the typical fibroblast-like morphology in P3 (Figure 1B). The surface screening of MSCs showed a positive expression for CD44, CD73, CD90, CD105, and CD166, and a negative expression for CD14, CD34, and CD45 antigens (Figure 1C). For MSCs, the FACS histogram plots are shown in supplementary Figure 1 (see Additional file 1: Figure S1), and their adipogenic, osteogenic, and chondrogenic potential was shown elsewhere [62]. The comparative flow-cytometric analysis of MSCs showed higher expression for their surface antigens compared with chondrogenic cells, as shown as a mean of three donors (see Additional file 2: Figure S5). Characterized MSCs were then induced to chondrogenic lineage differentiation. After 28 days of chondrogenic stimulation, the 6-μm-thick cryosections of the pellets showed cartilage-specific proteoglycan and were positive for Alcian blue staining (Figure 1D), in contrast to unstimulated control samples (Figure 1E). The chondrogenic ability of these samples was further ensured by the positive expression of cartilage-specific collagen type II (Figure 1F), compared with control samples (Figure 1G). On the gene level, the chondrogenic nature was verified by the expression of cartilage-specific genes *COL2A1* and *SOX9*. Both genes showed significantly upregulated expression in the chondrogenic samples compared with undifferentiated MSCs and controls (Figure 1H). These results confirmed the well-advanced state of chondrogenic differentiation.

### Isolation of chondrogenic differentiated cells

**Figure 2:**
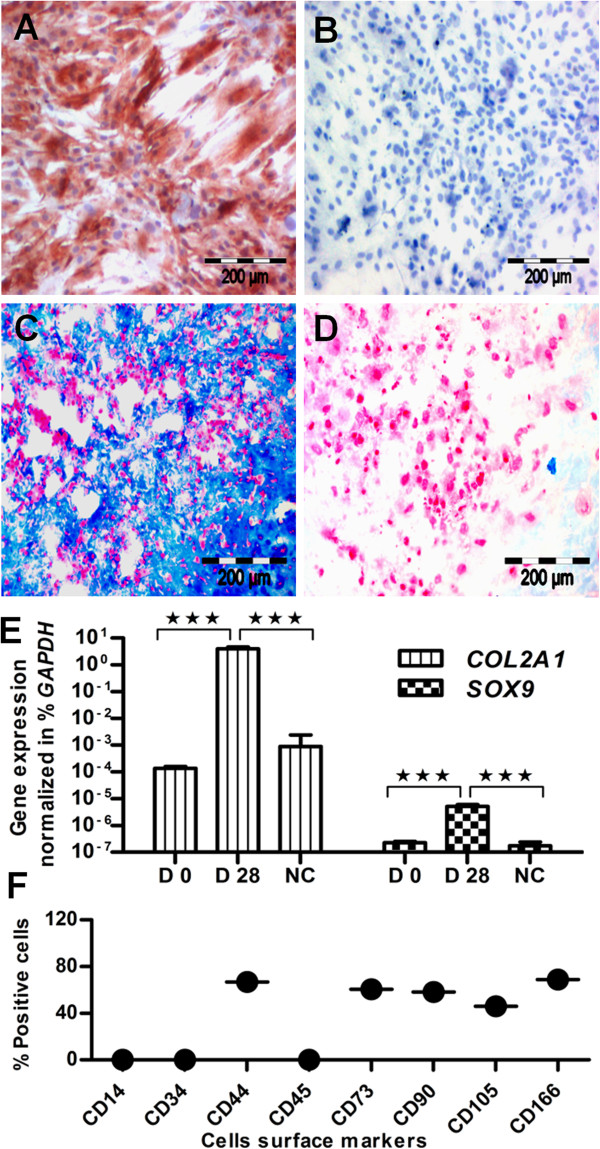
**Isolation of chondrogenically differentiated cells and their confirmation.** Chondrogenically differentiated cells were isolated from chondrogenic pellets. **(A)** Their chondrogenic character showed a positive collagen type II, **(C)** and Alcian blue staining, whereas the controls were negative for both **(B)** collagen type II **(D)** and Alcian blue staining. **(E)** After 28 days of induction, the chondrogenically differentiated cells showed significantly upregulated expression for cartilage-specific genes like *COL2A1* and *SOX9* compared with controls and undifferentiated cells, day 0 (*n* = 3), mean ± SEM. **(F)** Flow-cytometric analysis showed a relatively lower expression of CD44, CD73, CD90, CD105, and CD166 antigens compared with undifferentiated MSCs (*n* = 3). Student *t* test was performed for statistical analysis, and asterisks were assigned in the order P* < 0.05, P** < 0.01, and P*** < 0.001, mean ± SEM. Bar, 200 μm.

After 28 days of chondrogenic differentiation, the cells were isolated from the compact pellets with enzymatic cues consisting of 300 U of collagenase II, 20 U of collagenase P, and 2 m*M* CaCl_2_ (Figure 2) [59]. After successful isolation, the cells were cultivated in culture flasks to remove the components of the extracellular matrix, and their differentiated state was maintained in the presence of chondrogenic differentiation stimulus TGF-β3 for 14 days, and 2 × 10^5^ cells were transferred to chamber slides for histologic and immunohistochemical assessment. The chondrogenic potential of cultured cells showed a positive expression of collagen type II (Figure 2A) compared with control samples (Figure 2B). Similarly, the chondrogenic-stimulated samples were positive for Alcian blue staining (Figure 2C) compared with unstimulated control samples (Figure 2D), indicating cartilage-specific proteoglycan in the culture. This indicates that the cell-isolation procedure, removal of extracellular matrix, and subsequent cultivation does not affect the chondrogenic potential of cultured cells.

Similarly, on the gene level, they showed significantly higher expression of *COL2A1* and *SOX9* compared with undifferentiated MSCs and unstimulated controls (Figure 2E). After removing the extracellular components, the cultured cells were isolated with trypsinization (after 3 days) and subsequently analyzed for surface antigens. The surface screening showed a reduced expression for CD44, CD73, CD90, CD105, and CD166 antigens (Figure 2F; see Additional file 3: Figure S2) compared with undifferentiated MSCs (Figure 1H), indicating that MSCs reduce their expression for above-surface CD epitopes on chondrogenic differentiation. This statement is further confirmed by the comparative flow-cytometric analysis of surface antigens for three independent donors, in chondrogenically differentiated cells compared with undifferentiated and dedifferentiated cells (see Additional file 2: Figure S5).

### Dedifferentiation of chondrogenic differentiated cells

**Figure 3:**
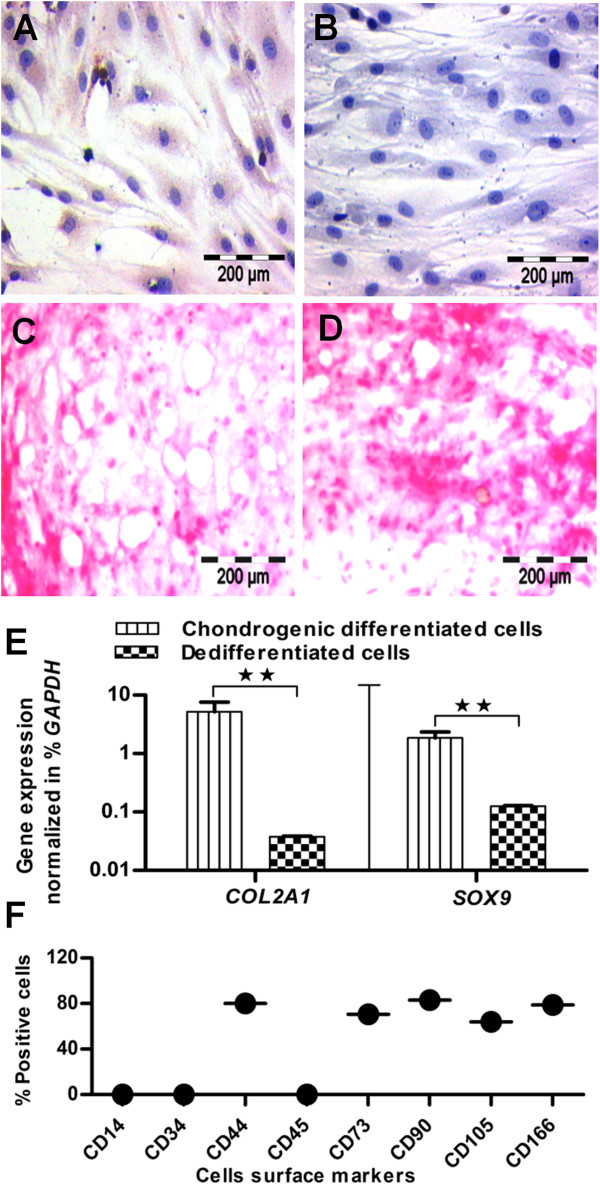
**Dedifferentiation of chondrogenically differentiated cells.** Chondrogenically differentiated cells were converted to dedifferentiated cells **(A)**, which showed almost the same collagen type II expression as **(B)** in control samples, and similarly **(C)** uniform expression for Alcian blue staining, **(D)** like their unstimulated control samples. **(E)** On the gene level, dedifferentiated cells showed significantly downregulated expression of *COL2A1* and *SOX9* genes, and confirmed the well-advanced state of dedifferentiation (*n* = 3). **(F)** Flow-cytometric profile of CD markers showed a relatively lower expression for dedifferentiated cells compared with the undifferentiated state of MSCs (n = 3), Student *t*test was performed for statistical analysis, and asterisks were assigned in the order P* < 0.05, P** < 0.01, and P*** < 0.001; mean ± SEM. Bar, 200 *μ*m.

To measure the chemotaxis potential for different states of MSCs, the isolated chondrogenically differentiated cells were dedifferentiated in the MSC culture/expansion medium, to generate their dedifferentiated progeny. After five passages, they showed intensive proliferation and converted into dedifferentiated progenitor cells. To inspect whether the chondrogenic character is still present in dedifferentiated cells, we examined them for collagen type II expression. The collagen type II staining was almost as negative as that of control samples (Figure 3A and B). Similar to control samples, they showed negative Alcian blue staining (Figure 3C, D), indicating the absence of proteoglycan in dedifferentiated cells. On the gene level, they showed significantly downregulated expression of *COL2A1* and *SOX9* genes compared with chondrogenically differentiated cells (Figure 3E). In conclusion, these results confirmed the well-advanced state of dedifferentiation. Surface analysis of CD antigens again showed higher expression for CD44, CD73, CD90, CD105, and CD166 (Figure 3F; see Additional file 4: Figure S3) compared with chondrogenic differentiated cells, but this is relatively lower than MSC expression. Conclusively, the surface CD profile of MSCs changed with differentiation, but was not completely recovered after dedifferentiation, as confirmed by the quantitative measurement of surface antigens of three independent donors (see Additional file 2: Figure S5).

### Migration of undifferentiated, chondrogenic differentiated, and dedifferentiated cells in response to serum-mediated chemotaxis

**Figure 4:**
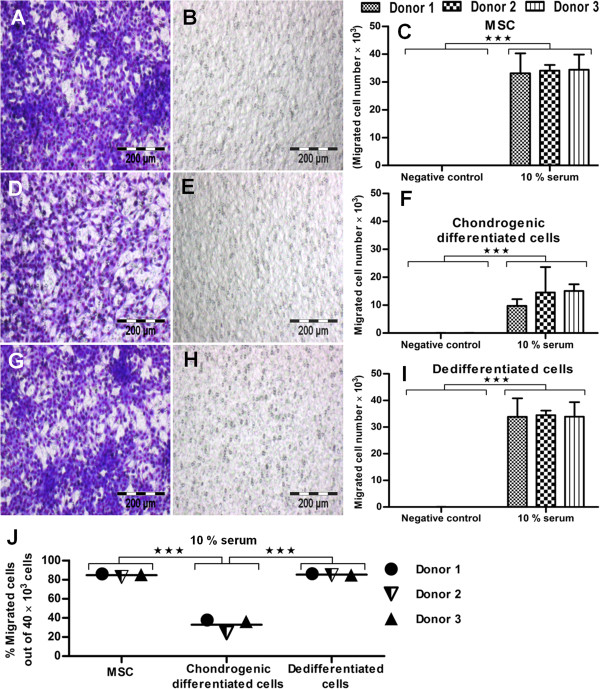
**Serum-mediated chemotaxis for undifferentiated, chondrogenic differentiated, and dedifferentiated states of MSCs. (A)** Serum-mediated chemotaxis showed higher hemacolor staining for MSCs compared with **(B)** control. **(C)** Quantification assessment with Image J software revealed more migrated cells for undifferentiated MSCs compared with control. **(D)** Chondrogenically differentiated cells showed intermediate hemacolor staining compared with **(E)** control, and **(F)** quantification assessment confirmed their low migration potential (33%). **(G)** Similarly, dedifferentiated cells again showed intense hemacolor staining compared with **(H)** control, **(I),** and the number of migrated cells was confirmed with Image J software. **(J)** The percentage of migrated cells relative to total cell number (40 × 10^3^) is shown for undifferentiated, chondrogenic differentiated, and dedifferentiated states of MSCs (*n* = 3). Student *t* test was performed for statistical analysis, and asterisks were assigned in the order P* < 0.05, P** < 0.01, and P*** < 0.001, mean ± SEM. Bar, 200 *μ*m.

After generating different states of MSCs (undifferentiated, chondrogenic differentiated, and their derived dedifferentiated state), we performed the chemotaxis assay to determine the relative effect of cell migration on these states. As stem cells-guided migration is considered one crucial parameter among all preclinical characterizing parameters [54,60]. Therefore, migration potential was analyzed with 10% allogenic serum for undifferentiated MSCs, chondrogenic differentiated, and dedifferentiated cells. Undifferentiated MSCs showed intensive hemacolor staining for migrated cells (Figure 4A) compared with control samples (Figure 4B). The quantification-assessment tool of Image J software confirmed the migration of about 3.3 × 10^4^ cells for the undifferentiated state of MSCs (Figure 4C). Similarly, the chondrogenically differentiated cells showed less hemacolor staining for migrated cells (Figure 4D) compared with the undifferentiated state of MSCs (Figure 4A). The control samples were negative (Figure 4E). The quantification assessment confirmed the migration of about 1.3 × 10^4^ cells for the chondrogenic differentiated state of MSCs (Figure 4F), suggesting that the differentiated state limits the rate of cell migration. On subsequent dedifferentiation, the cells again showed higher hemacolor staining (Figure 4G) versus chondrogenic cells (Figure 4D) for migrated cells. The control samples showed negligible cell migration (Figure 4H). The quantitative calculation ensured the migration of about 3.4 × 10^4^ cells for the dedifferentiated state of MSCs (Figure 4I).

In conclusion, the percentage comparison relative to total cell number (40 × 10^3^) revealed about 33%, 84%, and 85% cell migration for the chondrogenic differentiated, undifferentiated, and dedifferentiated states of MSCs, respectively (Figure 4J). This is in line with the statement that mature chondrocytes have relatively low migration potential *in vivo* for cartilage repair, because of its inherent architectural nature [20,22]. Allogenic 10% serum-mediated chemotaxis recruited relatively more cells for the undifferentiated (84%) and dedifferentiated states (85%), compared with the chondrogenic differentiated state (33%) of MSCs.

### Migration of undifferentiated, chondrogenic differentiated, and dedifferentiated cells in response to CCL25-mediated chemotaxis

**Figure 5:**
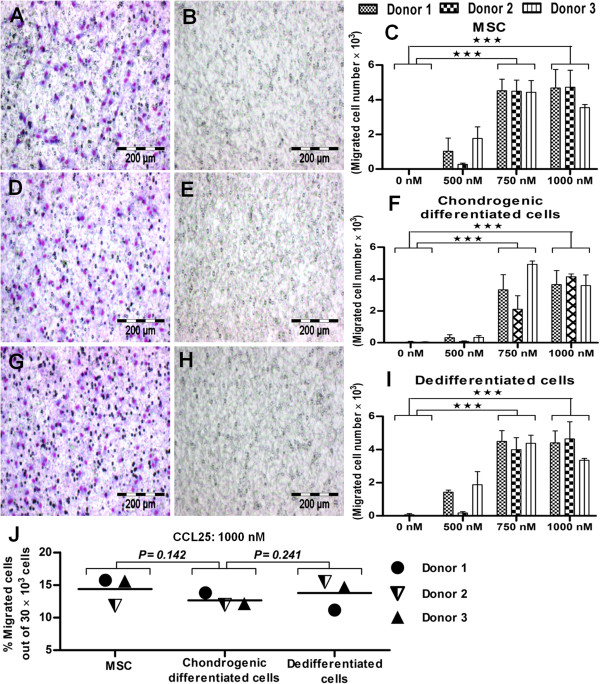
**CCL25-mediated chemotaxis for undifferentiated, chondrogenic differentiated, and dedifferentiated states of MSCs. (A)** In CCL25-mediated chemotaxis, the cell-migration assay showed almost uniform hemacolor staining for undifferentiated **(D),** chondrogenic differentiated **(G)**, and dedifferentiated states of MSCs compared with their **(B**, **E**, and **H)** respective controls. **(F)** Quantification assessment with Image J software confirmed a relatively low level of migration for chondrogenically differentiated cells (12%) compared with **(C)** undifferentiated and **(I)** dedifferentiated states of MSCs. **(J)** The percentage migration analysis relative to total cell number (30 × 10^3^) is given for undifferentiated, chondrogenic differentiated, and dedifferentiated states of MSCs (*n* = 3). Student *t* test was performed for statistical analysis, and asterisks were assigned in the order P* < 0.05, P** < 0.01, and P*** < 0.001, mean ± SEM. Bar, 200 *μ*m.

Biochemically, the serum is a complex and an undefined cue for diverse known and unknown functions, including migration [39-42]. Hence, it emphasizes the need for a known chemokine for guided and targeted cell migration. Moreover, CCL25 is an important chemoattractant and well known to initiate the process of inflammation, cellular mobilization, and migration of cells for effective regeneration [63,64]. In this scenario, CCL25 has been tested by our group [44,54] and reported as an important chemokine for targeted stem cell migration in regenerative medicine. Therefore, we assessed the relative effect of cell migration on different states of MSCs (undifferentiated, differentiated, and dedifferentiated), by using different concentrations of CCL25 (500 n*M*, 750 n*M*, and 1,000 n*M*). At 1,000 n*M* concentration of CCL25, we found almost uniform hemacolor staining for undifferentiated (Figure 5A), chondrogenic differentiated (Figure 5D), and dedifferentiated states of MSCs (Figure 5G) compared with their respective controls (Figure 5B, E, and H). The quantification assessment for different states of MSCs was performed with Image J software. For different concentrations of CCL25 (500 n*M*, 750 n*M*, and 1,000 n*M*), the quantitative analysis confirmed the differences in cell migration for different states of MSCs. For instance, at 1,000 n*M* concentration, about 3.8 × 10^3^ cells showed migration for the chondrogenic differentiated state (Figure 5F), about 4.5 × 10^3^ cells for the undifferentiated state (Figure 5C), and about 4.4 × 10^3^ cells for the dedifferentiated state (Figure 5I) of a total of 30 × 10^3^ cells for each state of MSCs.

To show an obvious comparative representation of cell migration in different states of MSCs, percentage quantification analysis was performed. The percentage quantification assessment with Image J software revealed a cell migration of about 12% for the chondrogenic differentiated state, about 14% for the undifferentiated state, and about 13% for the dedifferentiated state of MSCs of a total 30 × 10^3^ cells (Figure 5J) in response to CCL25-mediated chemotaxis at a concentration of 1,000 n*M* chemokine.

In conclusion, chondrogenically differentiated cells showed a low migration potential compared with undifferentiated MSCs and dedifferentiated cells in response to serum-mediated chemotaxis. The reason for this low migration potential of chondrogenic differentiated cells may be hidden in the loss/modification of migration-specific receptors to serum, during differentiation. Conversely, chondrogenically differentiated cells had almost equally migrated (12%) compared with undifferentiated MSCs (14%) and dedifferentiated cells (13%) in response to CCL25-mediated chemotaxis. This enhances the value of CCL25 as a guided chemokine for cartilage repair, as its chemotactic activity is not influenced by the differentiated or undifferentiated nature of the cells. Alternatively, the receptors taking part in the CCL25-mediated chemotaxis, perhaps had not lost/modified their expression, during chondrogenic differentiation. However, CCL25 controls cellular trafficking irrespective of the cell architectural nature and the differentiated state, so it is important to investigate CCL25 as a migratory cue, in a broad spectrum, as the concentration of CCL25 (1,000 n*M*) was maintained similarly for undifferentiated, differentiated, and dedifferentiated states of MSCs.

Why did the chondrogenic differentiated state show an almost equal rate of cell migration in CCL25-mediated chemotaxis compared with undifferentiated and dedifferentiated states of MSCs? CCR9, a binding receptor of CCL25 chemokine, was investigated to determine the role in guided cell migration.

### Analysis of CCR9, a cellular receptor of CCL25 chemokine

**Figure 6:**
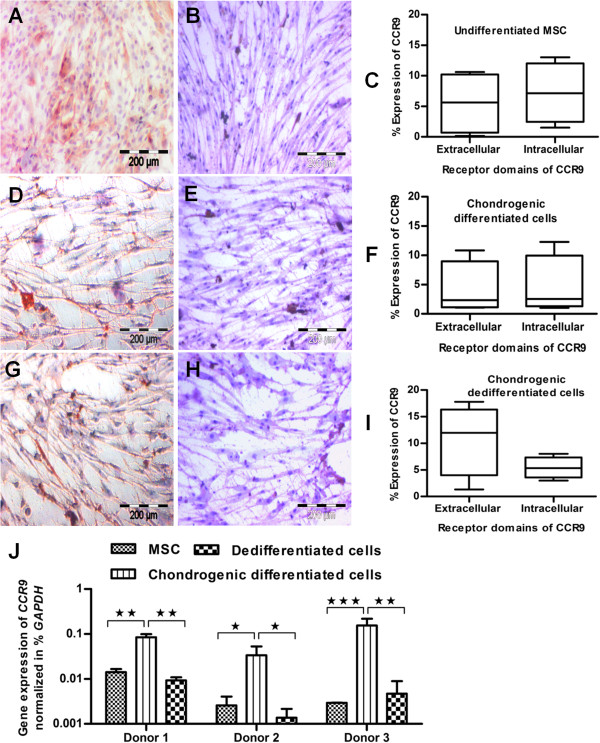
**Analysis of CCR9, a cellular receptor of CCL25 chemokine.** The immunohistochemical analysis of CCR9 showed greater staining for **(A)** the undifferentiated state compared with **(D)** chondrogenic differentiated and **(G)** dedifferentiated states of MSCs. The percentage flow-cytometric measurement for extracellular and intracellular domains of CCR9 is given in the form of average (*n* = 3) with a horizontal line, and bars represents a minimum or maximum expression for **(C)** undifferentiated state, **(F)** chondrogenic differentiated, and **(I)** dedifferentiated states of MSCs. **(J)** On the gene level, the qPCR analysis revealed significantly upregulated expression for *CCR9* receptor in the chondrogenic differentiated state compared with undifferentiated and dedifferentiated states of MSCs (*n* = 3). Student *t* test was performed for statistical analysis, and asterisks were assigned in the order P* < 0.05, P** < 0.01, and P*** < 0.001, average ± SD. Bar, 200 *μ*m.

CCL25 is an identified chemokine for stem cells-targeted migration, and CCR9 is its known receptor [54,57,58]. Thus, the undifferentiated, chondrogenic differentiated, and dedifferentiated states of MSCs were examined for CCR9 expression. The undifferentiated state of MSCs showed a bit greater and homogeneous staining for CCR9 receptor (Figure 6A) compared with the chondrogenic differentiated (Figure 6D) and dedifferentiated states (Figure 6G). The corresponding controls were negative for all states of MSCs (Figures 6B, E, and H). This indicates the presence of CCR9 receptor in only undifferentiated, differentiated, and dedifferentiated states of MSCs on a qualitative basis.

For quantitative measurement, flow-cytometric analysis was performed to examine the expression level of CCR9 receptor. However, CCR9 receptor has two distinct domains called extracellular and intracellular; thus flow-cytometric analysis was performed to screen all states of MSCs for its two domains. After analysis, their expression was quantitatively expressed in the form of averages, along with standard deviations (*n* = 3); here the representative bars are divided to specify a minimum or maximum expression. For undifferentiated (Figure 6C) and chondrogenic differentiated (Figure 6F) states of MSCs, the expression of extracellular and intracellular domains was almost similar and about 10% for each domain, whereas for the dedifferentiated state of MSCs, the expression of extracellular and intracellular domains was about 18% and 5%, respectively (Figure 6I). The detailed histogram plots for extracellular and intracellular domains of CCR9 receptors are in the form of supplemental files, for undifferentiated, chondrogenic differentiated, and dedifferentiated states of MSCs (see Additional file 5: Figure S4).

The expression level of CCR9 was almost homogeneous for extracellular (10%) and intracellular (10%) domains, and collectively about 20% for each undifferentiated and chondrogenic differentiated state of MSCs, and a bit higher, about 23%, for their dedifferentiated state (Figure 6C, F, and I). On a molecular level, the qPCR analysis was performed for *CCR9* gene expression, which showed significantly upregulated expression for the chondrogenic differentiated state compared with undifferentiated and dedifferentiated states of MSCs (Figure 6J). Here, the protein and gene level expression of *CCR9* confirms the presence of receptor in undifferentiated, differentiated, and dedifferentiated states of MSCs.

## Discussion

Guided stem cell migration is a vital approach for cell recruitment to the point of injury or damage. For cartilage regeneration, the endogenous mobilization of chondrogenic cells and subsequent migration could be a promising approach. In this context, the *in vivo* model remains to be proven; however, we performed some primary experiments *in vitro* for guided cell migration of chondrogenic differentiated cells, a step toward the search for the right cells for the job. Cartilage tissue is a combination of progenitor and differentiated cell types, such as chondroblasts, chondrocytes, and dedifferentiated cells [20,29,32]. Therefore, in the current study, we analyzed the chemotactic ability of undifferentiated, chondrogenic differentiated, and dedifferentiated states of MSCs with serum- or CCL25-mediated chemotaxis. Moreover, the surface profile of CD markers was investigated to screen and specify the chondrogenic differentiated state on the basis of typical MSC antigens. The flow-cytometric analysis revealed a low level of expression for CD44, CD73, CD90, CD105, and CD166 antigens in the chondrogenic differentiated state compared with undifferentiated and dedifferentiated states of MSCs, suggesting that chondrogenically differentiated cells reduce their expression for these surface antigens. In addition, these surface antigens are good characterizing markers for chondrogenically differentiated cells and their progeny, and in line with previously published reports, recommending the use of such surface markers for identification of chondrogenic cells [65,66].

Chondrogenically differentiated cells were assessed for cell migration in response to 10% allogenic serum-mediated chemotaxis [39] or an established concentration of 500 n*M*, 750 n*M*, and 1,000 n*M* CCL25-mediated chemotaxis [44,54]. In serum-mediated chemotaxis, we observed a significant decrease in recruited cells for the chondrogenic differentiated state (33%) compared with undifferentiated (84%) and dedifferentiated states (85%) of MSCs. We would recommend the use of chondrogenically differentiated cells for therapeutic repair of cartilage, as they have active signaling pathways, chondrogenic character, and biological paradigms of the differentiated state. For fast-track regeneration, the cartilaginous nature and chemokinetic ability of chondrogenically differentiated cells could be a beneficial asset.

For migration, serum is considered a very good chemoattractant for recruitment of cells; however, its composition is very complex, and its role as yet unknown in several biologic functions [40-42]. Therefore, we applied CCL25 for cell recruitment, a well-known chemokine for targeted stem cell migration [44,54]. Here, we noticed negligibly low migration potential for chondrogenically differentiated cells compared with serum-mediated chemotaxis.

In serum-mediated chemotaxis, we blame the inherent architectural ability of the chondrogenic differentiated state of MSCs for its limited cell migration. As chondrocytes have limited mitotic potential, lack of innervations and vascular supply, and are entrapped in the extracellular matrix, almost no physical contact with each other and restricted migration potential to the point of injury *in vivo*[20-22]. The CCL25-mediated chemotaxis has recruited 14%, 13%, and 12% of cells, respectively for undifferentiated, dedifferentiated, and chondrogenic differentiated states of MSCs. Here, the migration rate of chondrogenically differ-entiated cells was almost similar to the differentiated or undifferentiated states of MSCs. Perhaps the activation of some receptors and signaling in the chondrogenic differentiated state is the cause of this higher chemotactic ability. In this way, CCL25-mediated chemotaxis favors guided cellular trafficking, and we recommend the use of CCL25 as a migratory cue in regenerative applications. This especially highlights the significant use of chondrogenically differentiated cells for cartilage restoration because they have almost the same migration potential compared with the undifferentiated and dedifferentiated states of MSCs. The collective use of CCL25 chemokine and chondrogenic differentiated states of MSCs could be more beneficial for cartilage regeneration, possibly because of their active signaling, so the use of chondrogenically differentiated cells for cartilage repair could be fruitful.

We propose that chemotactic signals and inflammatory response from injured sites could induce cellular mobilization and create an intermediate pore sizes for subsequent movement of cells to the injured sites. Our suggestion is in line with the reports that inflammation plays a critical role in the regeneration of cartilage tissue [32,67-70].

Cytokine- and especially chemokine-based migration is a crucial step for *in vivo* regenerative application [43,71]. In this context, CCL25/CCR9 is a chemokine/receptor pair and plays a key regulative role in stem cell migration [57,58]. In our study, the immunohistochemical analysis was performed for the assessment of CCR9 receptor, which showed almost uniform staining for undifferentiated, differentiated, and dedifferentiated states of MSCs, indicated the presence of receptors in these states. However, we have not used the positive controls for immunohistochemical staining of CCR9 receptor, which is a study limitation. For quantitative analysis, flow-cytometric analysis was performed to measure the expression of the extracellular and intracellular domains of the CCR9 receptor. It has been reported that intracellular signaling is required for CCL25 activation and stimulation of chemoattractant ability [45,72]. The expression of extracellular and intracellular domains of CCR9 collectively revealed about 20% expression for the undifferentiated and chondrogenic differentiated states, but about 23% expression for the dedifferentiated state of MSCs.

On the molecular level, we analyzed all states of MSCs for *CCR9* gene expression by performing qPCR, to identify the normalized amount of receptor to *GAPDH*[73]. Gene analysis showed significantly upregulated expression of *CCR9* in the chondrogenic differentiated state compared with the undifferentiated and dedifferentiated states of MSCs. To correlate the protein- and gene-level expression, we propose that any apparent observational change in the protein and gene level of the CCR9 receptor could be the cause of posttranscriptional and posttranslational level modification. In some cases, the protein-level expression of CCR9 does not correlate with mRNA level expression, and such noncorrelation of CCL25/CCR9 has been reported in mucosal immune systems on protein and gene levels [57], and favors our speculation about posttranslational modification. Furthermore, it also supports the Monte Carlo effect, a hypothesis drawn about the biological importance that the level of mRNA expression is not always directly correlated with the protein expression [74]. In addition, the chemotactic ability of CCL25 not only is the result of a cellular receptor of CCR9, but also is receiving signals from other receptors and signaling cascades for activation, stimulation, and cellular migration [64].

The current study generated the knowledge of the comparative surface CD profile, chemotaxis, and migration potential for undifferentiated, chondrogenic differentiated, and dedifferentiated states of MSCs. To understand the molecular mechanisms of migration in different states of MSCs could be valuable to identify the potential targets for wound healing, damage repair, and regeneration.

## Conclusions

The chemokines, cytokines, and growth factors in consequence of inflammation facilitate cells homing to the site of injury and improve tissue regeneration [75]. These regenerative strategies emphasize the importance of targeted and guided chemotaxis for cell migration. Therefore, the chondrogenically differentiated cells were investigated for their chemotactic ability. In this context, chondrogenic pellets were generated from MSCs by using chondrogenic differentiation medium for 28 days. The chondrogenic nature of the pellets was confirmed by proteoglycan-specific Alcian blue staining, cartilage-specific collagen type II staining, and significantly upregulated cartilage-specific genes *COL2A1* and *SOX9*. Then differentiated cells were isolated from the intact chondrogenic pellets with enzymatic cues consisting of 300 U of collagenase II, 20 U of collagenase P, and 2 m*M* CaCl_2_[59]. After successful isolation, the differentiated cells were again verified for chondrogenic features, and they were positive for Alcian blue staining, collagen type II staining, and showed an upregulated expression of *COL2A1* and *SOX9*.

Afterward, the chondrogenically differentiated cells were washed with PBS, and extracellular matrix was removed; then their surface was analyzed for surface CD antigens. The surface profile of chondrogenically differentiated cells showed a positive expression of CD44, CD73, CD90, CD105, and CD166, but notably this expression was about 40% to 50% lower than that in undifferentiated and dedifferentiated states of MSCs. In serum-mediated chemotaxis, the number of migrated cells was significantly lower for the chondrogenic differentiated state (33%) compared with the undifferentiated (84%) and dedifferentiated (85%) states of MSCs, of a total 40 × 10^3^ cells. In CCL25-mediated chemotaxis, the number of migrated cells was almost the same for the chondrogenic differentiated state (12%) compared with undifferentiated (14%) and dedifferentiated states (13%) of MSCs, of a total of 30 × 10^3^ cells.

The expression of CCR9 was examined with immunohistochemistry and flow-cytometric analysis, which confirmed the presence of CCR9 in undifferentiated, differentiated, and dedifferentiated states of MSCs. On the molecular level, the expression of *CCR9* was significantly upregulated in the chondrogenic differentiated state compared with the undifferentiated and dedifferentiated states of MSCs. We propose that CCL25-mediated chemotaxis is influenced by the expression of CCR9 and stimulates guided cell migration in all states of MSCs. Cell migration as a result of mutual interaction of CCL25 and CCR9 has already been studied [57], and supports our conclusive message of guided chemotaxis. Moreover, the coupling interactions between CCL25 and CCR9 induce cell migration in porcine mucosal tissue and in the immune system during fetal development [57,58].

The *in vivo* migration of chondrogenically differentiated cells remains to be proven; however, *in vitro* oriented cell migration and homing study could provide valuable arguments in this direction for further investigation.

## Abbreviations

CaCl2: Calcium chloride; CCL25: Chemokine (C-C motif) ligand 25; CCR9: C-C chemokine receptor type-9; CD: Cluster of differentiation; COL2A1: Collagen type IIα1; FACS: Fluorescence-activated cell sorting; GAPDH: Glyceraldehyde-3-phosphate dehydrogenase; MSC: Mesenchymal stem cell; PBS: Phosphate-buffered saline; PCR: Polymerase chain reaction; SOX9: *SRY (sex-determining region Y)-box-9*; TGF: transforming growth factor.

## Competing interests

Michael Sittinger is a shareholder of CellServe Ltd. (Berlin, Germany) and BioRetis Ltd. (Berlin, Germany) and works as consultant for BioTissue Technologies Ltd. (Freiburg, Germany), which develops tissue transplants for the regeneration of bone and cartilage. The product activities of the companies are not related to the scientific topics presented here. The other authors indicate no potential conflict of interest. All authors disclose any financial and personal relationship with people or organizations that could inappropriately influence this scientifically oriented *in vitro* study. Therefore, no competing financial interests exist.

## Authors’ contributions

MU performed experiments, participated in the design and coordination of the study, and prepared the primary draft of the manuscript. MU, JR, and MS evaluated and cross checked the data, helped in the final drafting of manuscript, helped in processing of FACS data, and participated in the design and coordination of study. JE provided the bone marrow samples and helped in the coordination and final drafting of manuscript. All authors read and approved the final manuscript.

## Supplementary Material

Additional file 1: Figure S1 Flow-cytometric analysis of undifferentiated MSCs, isolated from bone marrow. MSCs in passage 3 (*n* = 3) were uniformly positive for typical surface markers like CD166, CD105, CD90, CD73, and CD44, as examples given for a single donor, and were negative for hematopoietic cell markers like CD45, CD34, and CD14. Click here for file

Additional file 2: Figure S5 Comparative flow-cytometric profile of undifferentiated, chondrogenic differentiated and dedifferentiated cells. The comparative surface profile of CD markers showed a lower expression for chondrogenically differentiated cells compared with undifferentiated and dedifferentiated cells. Generally the undifferentiated, differentiated, and dedifferentiated states of MSCs were positive for CD44, CD73, CD90, CD105, and CD166 and negative for CD14, CD34, and CD45. The Student *t* test was performed for statistical analysis, and asterisks were assigned in the order P* < 0.05, P** < 0.01, and P*** < 0.001, mean ± SEM. Red asterisks represent the statistical comparison of undifferentiated cells versus dedifferentiated cells, whereas black asterisks represent the statistical comparison of undifferentiated cells versus chondrogenic differentiated cells.
Comparative flow-cytometric profile of undifferentiated, chondrogenic differentiated and dedifferentiated cells. The comparative surface profile of CD markers showed a lower expression for chondrogenically differentiated cells compared with undifferentiated and dedifferentiated cells. Generally the undifferentiated, differentiated, and dedifferentiated states of MSCs were positive for CD44, CD73, CD90, CD105, and CD166 and negative for CD14, CD34, and CD45. The Student *t* test was performed for statistical analysis, and asterisks were assigned in the order P* < 0.05, P** < 0.01, and P*** < 0.001, mean ± SEM. Red asterisks represent the statistical comparison of undifferentiated cells versus dedifferentiated cells, whereas black asterisks represent the statistical comparison of undifferentiated cells versus chondrogenic differentiated cells. Click here for file

Additional file 3: Figure S2 Flow-cytometric analysis of chondrogenically differentiated cells, isolated from chondrogenic pellets. Differentiated cells (*n* = 3) were positive for typical surface markers like CD166, CD105, CD90, CD73, and CD44, as exemplary of a single donor, and were negative for hematopoietic cell markers like CD45, CD34, and CD14. However, their plot expressions were not uniform and showed variations; moreover, chondrogenically differentiated cells significantly reduced their expression (about 40% to 50%) for CD166, CD105, CD90, CD73, and CD44. Click here for file

Additional file 4: Figure S3 Flow-cytometric analysis of dedifferentiated cells. After dedifferentiation, the cells (*n* = 3) again showed higher expression for typical surface markers like CD166, CD105, CD90, CD73, and CD44, as examples of a single donor, and were negative for hematopoietic cell markers like CD45, CD34, and CD14. However, their plot expressions were not uniform and showed variations; moreover, dedifferentiated cells significantly recovered their expression compared with chondrogenically differentiated cells but still were relatively lower than undifferentiated MSCs. Click here for file

Additional file 5: Figure S4 Flow-cytometric analysis of CCR9 receptor. Undifferentiated, chondrogenic differentiated and dedifferentiated cells (*n* = 3) were analyzed for CCR9, which is a cognate receptor of CCL25 chemokine. For complete assessment, the CCR9 examination was divided into extracellular and intracellular analysis, as examples of a single donor, which showed relatively lower level of expression for undifferentiated MSCs compared with chondrogenic differentiated and dedifferentiated cells. Moreover, the expression-profile plots, especially for the intracellular domain, were flatter compared with the extracellular domains. Click here for file
