# Supplementary figures and images for: Mesenchymal stem cells and their chondrogenic differentiated and dedifferentiated progeny express chemokine receptor CCR9 and chemotactically migrate toward CCL25 or serum
Source: Stem Cell Res Ther. 2013 Aug 19;4(4):99. doi: 10.1186/scrt310 (PMC3854782; doi:10.1186/scrt310)

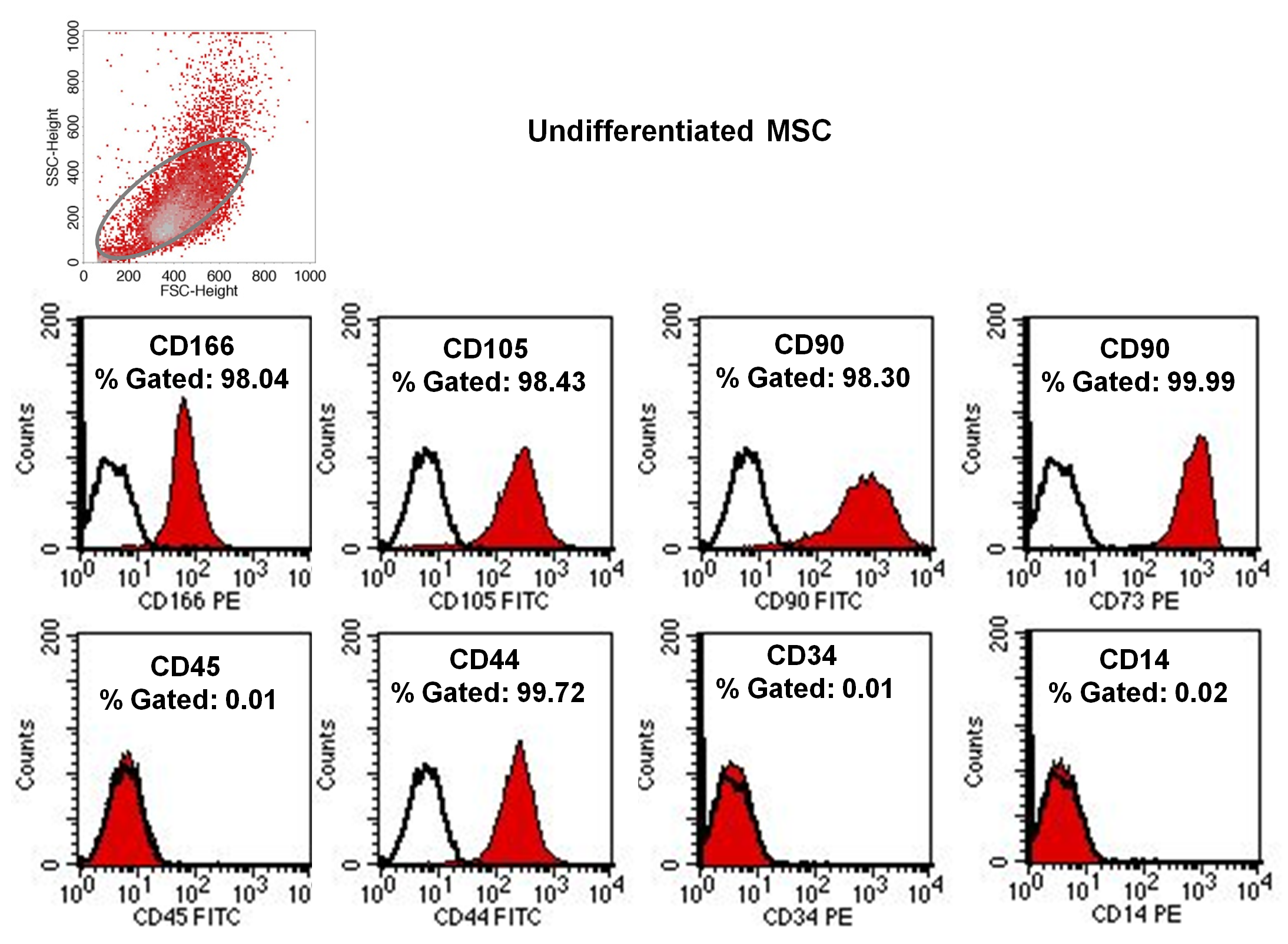

Supplement: Additional file 1: Figure S1 — Flow-cytometric analysis of undifferentiated MSCs, isolated from bone marrow. MSCs in passage 3 (n = 3) were uniformly positive for typical surface markers like CD166, CD105, CD90, CD73, and CD44, as examples given for a single donor, and were negative for hematopoietic cell markers like CD45, CD34, and CD14. [file scrt310-S1.tiff]

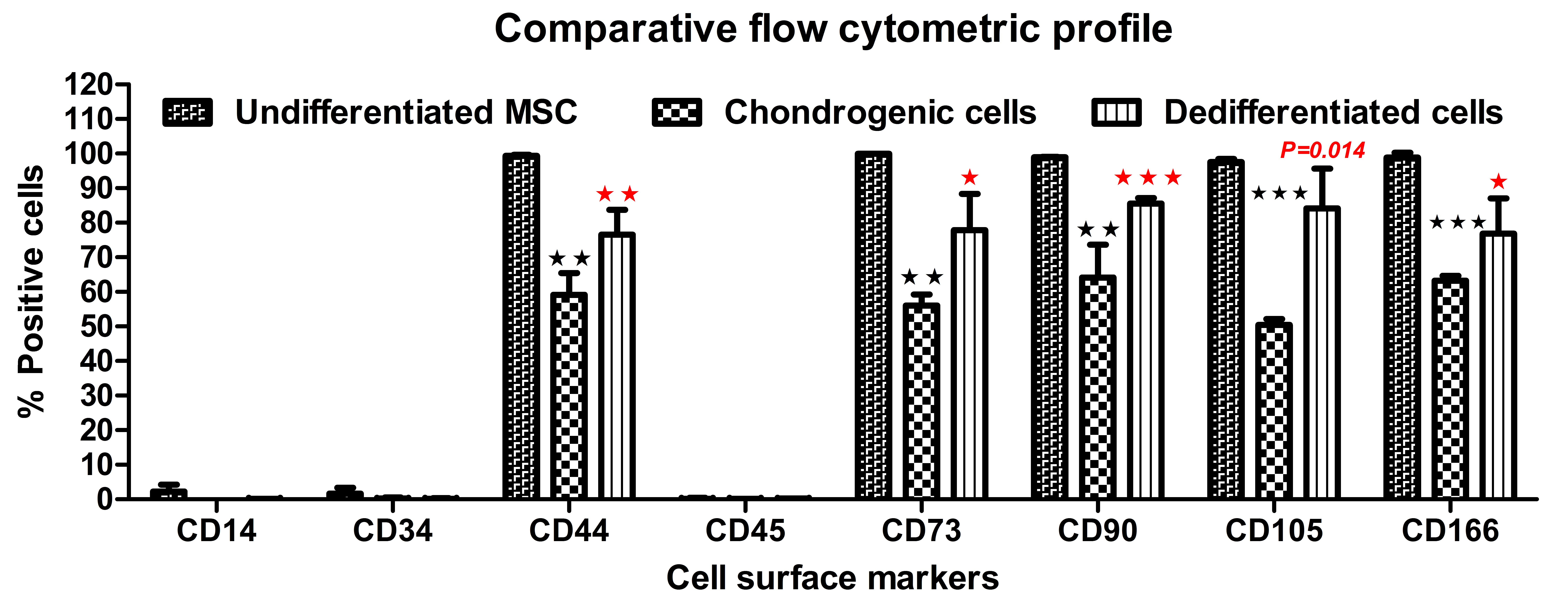

Supplement: Additional file 2: Figure S5 — Comparative flow-cytometric profile of undifferentiated, chondrogenic differentiated and dedifferentiated cells. The comparative surface profile of CD markers showed a lower expression for chondrogenically differentiated cells compared with undifferentiated and dedifferentiated cells. Generally the undifferentiated, differentiated, and dedifferentiated states of MSCs were positive for CD44, CD73, CD90, CD105, and CD166 and negative for CD14, CD34, and CD45. The Student t test was performed for statistical analysis, and asterisks were assigned in the order P* < 0.05, P** < 0.01, and P*** < 0.001, mean ± SEM. Red asterisks represent the statistical comparison of undifferentiated cells versus dedifferentiated cells, whereas black asterisks represent the statistical comparison of undifferentiated cells versus chondrogenic differentiated cells. Comparative flow-cytometric profile of undifferentiated, chondrogenic differentiated and dedifferentiated cells. The comparative surface profile of CD markers showed a lower expression for chondrogenically differentiated cells compared with undifferentiated and dedifferentiated cells. Generally the undifferentiated, differentiated, and dedifferentiated states of MSCs were positive for CD44, CD73, CD90, CD105, and CD166 and negative for CD14, CD34, and CD45. The Student t test was performed for statistical analysis, and asterisks were assigned in the order P* < 0.05, P** < 0.01, and P*** < 0.001, mean ± SEM. Red asterisks represent the statistical comparison of undifferentiated cells versus dedifferentiated cells, whereas black asterisks represent the statistical comparison of undifferentiated cells versus chondrogenic differentiated cells. [file scrt310-S2.tiff]

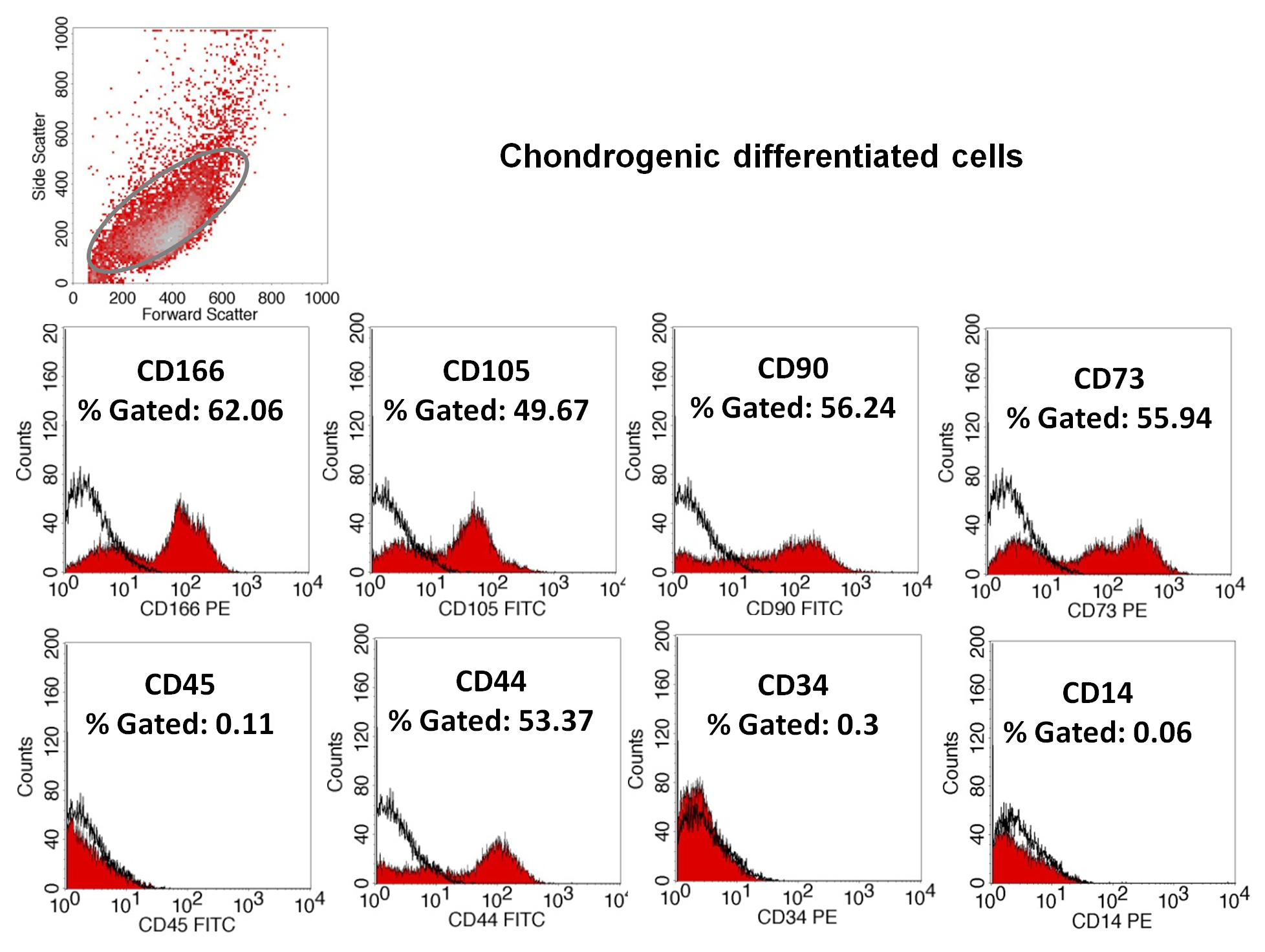

Supplement: Additional file 3: Figure S2 — Flow-cytometric analysis of chondrogenically differentiated cells, isolated from chondrogenic pellets. Differentiated cells (n = 3) were positive for typical surface markers like CD166, CD105, CD90, CD73, and CD44, as exemplary of a single donor, and were negative for hematopoietic cell markers like CD45, CD34, and CD14. However, their plot expressions were not uniform and showed variations; moreover, chondrogenically differentiated cells significantly reduced their expression (about 40% to 50%) for CD166, CD105, CD90, CD73, and CD44. [file scrt310-S3.tiff]

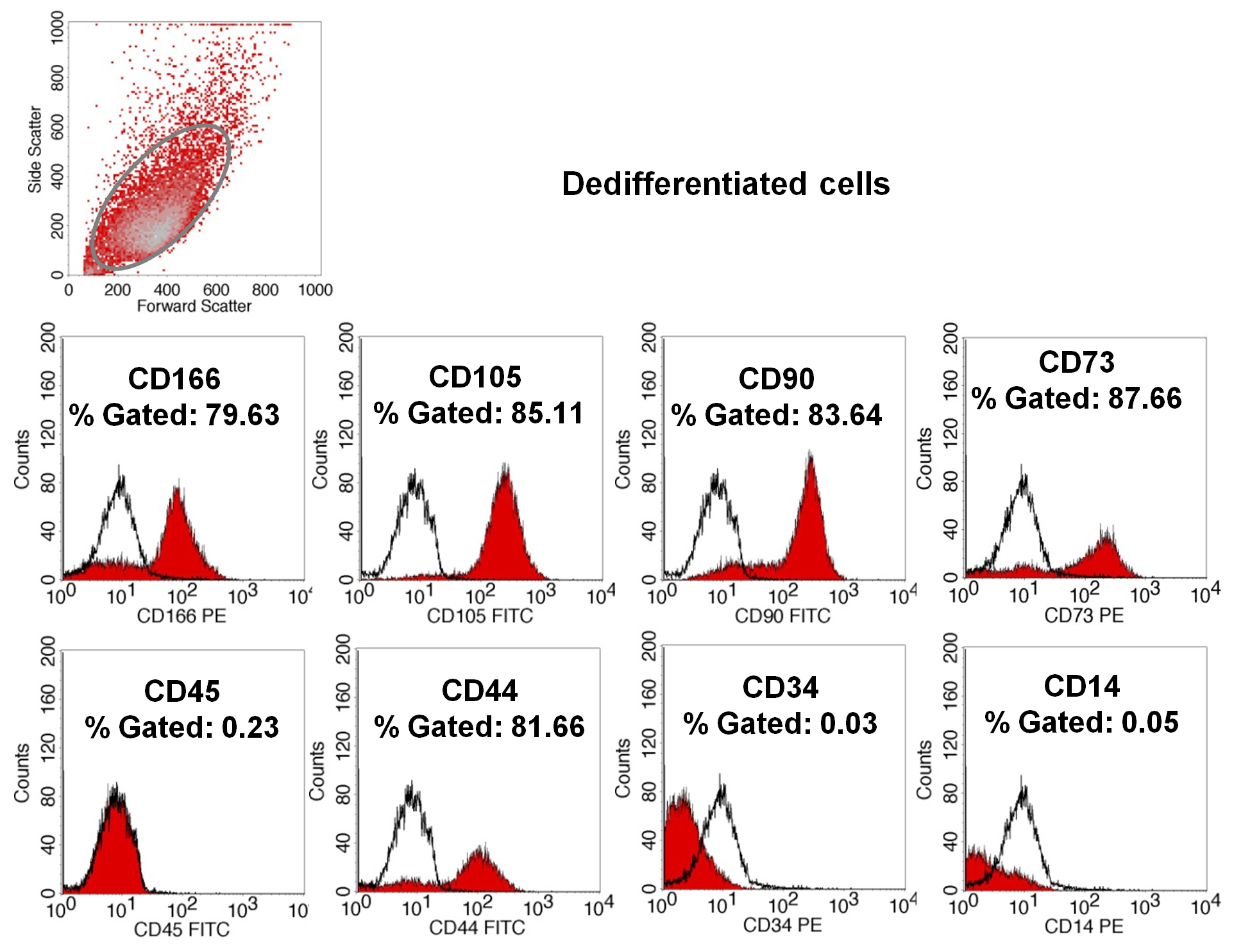

Supplement: Additional file 4: Figure S3 — Flow-cytometric analysis of dedifferentiated cells. After dedifferentiation, the cells (n = 3) again showed higher expression for typical surface markers like CD166, CD105, CD90, CD73, and CD44, as examples of a single donor, and were negative for hematopoietic cell markers like CD45, CD34, and CD14. However, their plot expressions were not uniform and showed variations; moreover, dedifferentiated cells significantly recovered their expression compared with chondrogenically differentiated cells but still were relatively lower than undifferentiated MSCs. [file scrt310-S4.tiff]

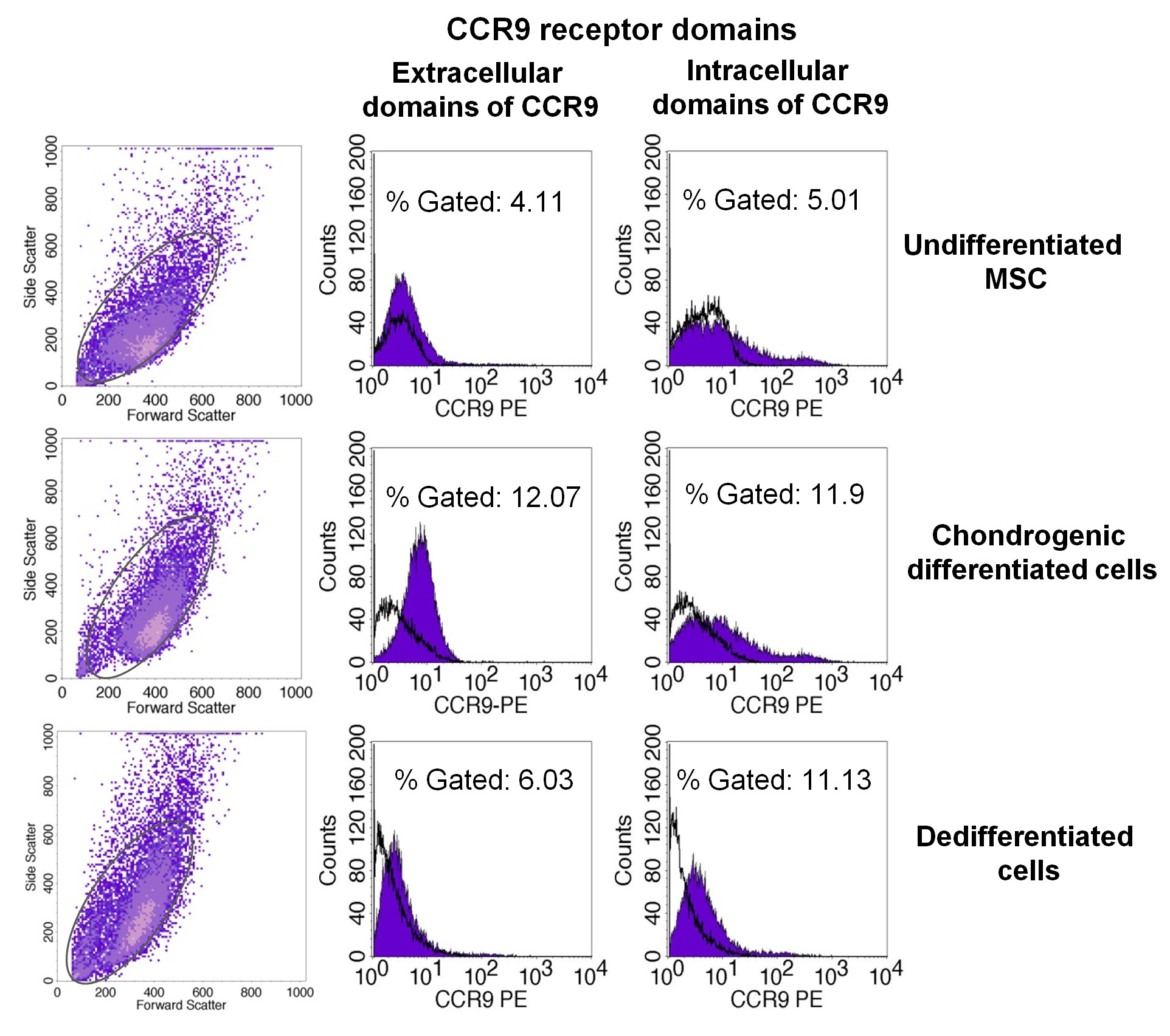

Supplement: Additional file 5: Figure S4 — Flow-cytometric analysis of CCR9 receptor. Undifferentiated, chondrogenic differentiated and dedifferentiated cells (n = 3) were analyzed for CCR9, which is a cognate receptor of CCL25 chemokine. For complete assessment, the CCR9 examination was divided into extracellular and intracellular analysis, as examples of a single donor, which showed relatively lower level of expression for undifferentiated MSCs compared with chondrogenic differentiated and dedifferentiated cells. Moreover, the expression-profile plots, especially for the intracellular domain, were flatter compared with the extracellular domains. [file scrt310-S5.tiff]
